# Central Activation of Alpha7 Nicotinic Signaling Attenuates LPS-Induced Neuroinflammation and Sickness Behavior in Adult but Not in Aged Animals

**DOI:** 10.3390/molecules26082107

**Published:** 2021-04-07

**Authors:** Elisa Navarro, Diana M. Norden, Paige J. Trojanowski, Jonathan P. Godbout, Manuela G. López

**Affiliations:** 1 Department of Pharmacology, Institute Teofilo Hernando for Drug Discovery, School of Medicine, Universidad Autónoma de Madrid, 28029 Madrid, Spain; elisa.navarro.gmesa@gmail.com; 2 Instituto de Investigación Sanitaria Hospital de la Princesa (IIS-IP), 28006 Madrid, Spain; 3 Department of Neuroscience, Institute for Behavioral Medicine Research, Center for Brain and Spinal Cord Repair, The Ohio State University Wexner Medical Center, Princeton, IL 61349, USA; diana.norden@gmail.com (D.M.N.); trojanowski.6@buckeyemail.osu.edu (P.J.T.)

**Keywords:** alpha7 nicotinic receptor, neuroinflammation, microglia, lipopolysaccharide, ageing

## Abstract

We previously reported that lipopolysaccharide (LPS) challenge caused microglial-mediated neuroinflammation and sickness behavior that was amplified in aged mice. As α7 nAChRs are implicated in the “Cholinergic anti-inflammatory pathway”, we aimed to determine how α7 nAChR stimulation modulates microglial phenotype in an LPS-induced neuroinflammation model in adult and aged mice. For this, BALB/c mice were injected intraperitoneally with LPS (0.33 mg/kg) and treated with the α7 nAChR agonist PNU282987, using different administration protocols. LPS challenge reduced body weight and induced lethargy and social withdrawal in adult mice. Peripheral (intraperitoneal) co-administration of the α7 nAChR agonist PNU282987 with LPS, attenuated body weight loss and sickness behavior associated with LPS challenge in adult mice, and reduced microglial activation with suppression of IL-1β and TNFα mRNA levels. Furthermore, central (intracerebroventricular) administration of the α7 nAChR agonist, even 2 h after LPS injection, attenuated the decrease in social exploratory behavior and microglial activation induced by peripheral administration of LPS, although this recovery was not achieved if activation of α7 nAChRs was performed peripherally. Finally, we observed that the positive results of central activation of α7 nAChRs were lost in aged mice. In conclusion, we provide evidence that stimulation of α7 nAChR signaling reduces microglial activation in an in vivo LPS-based model, but this cholinergic-dependent regulation seems to be dysfunctional in microglia of aged mice.

## 1. Introduction

There is increasing evidence indicating that inflammation is implicated in ageing and contributes to age-related neurodegenerative diseases, to the extent that it is considered the strongest factor for predicting longevity and neurodegeneration [1]. According to this hypothesis, ageing is characterized by a progressive increase in the pro-inflammatory status and by a reduction in the capability to cope with stressors, which would favor the disabilities associated with longevity [2].

Microglia are the resident innate immune cells within the central nervous system (CNS); they derive from monocyte progenitors that originate in the embryonic yolk sac and perform a variety of functions both in health and disease. Surveying microglia are constantly examining the microenvironment and have a ramified morphology. However, in response to pathology, microglia acquire an amoeboid morphology and actively migrate towards the site to clear pathogens or debris [3]. Moreover, microglia also perform other essential functions such as maintaining CNS homeostasis and plasticity, guarding and remodeling synapses [4,5,6]. 

During ageing, microglia acquire a “primed or reactive” phenotype. Primed microglia express increased pro-inflammatory markers including complement receptor 3 (CD11b), major histocompability complex II (MHCII), Toll-like receptors (TLRs) or pro-inflammatory cytokines such as IL-1β, IL-6 or TNF-α [7,8,9,10]. Related to these changes, the primed phenotype is also associated with a de-ramified and ameboid morphology similar to the activated state of microglia [11,12,13]. Why microglia adopt this primed phenotype during ageing remains unknown, although there is some evidence indicating that it may be caused by alterations in several regulatory pathways. For example, aged mice fail to increase IL-4R expression after LPS challenge and thus, the anti-inflammatory cytokine IL-4 is unable to return microglia to an M2 phenotype [14]. In addition, other regulatory systems that are altered during ageing include IL-10, CX_3_CL1, TGF-β or CD200, and their receptors [7,9,15,16,17]. Finally, additional factors that may contribute to the primed phenotype are oxidative stress, changes in microRNA expression and increased inflammasome activation [12]. Importantly, this primed phenotype has relevant pathophysiological consequences. In fact, the inflammatory response following peripheral or central immune activation is prolonged and exacerbated in aged animals [8] and this exaggerated inflammatory response is associated with behavioral alterations such as increased depressive-like behavior [18], prolonged sickness behavior [8] and cognitive impairment [19]. Furthermore, given the importance of microglia in CNS functioning and its alterations reported during ageing, it is not surprising that a great number of microglia-related genes have been associated with neurodegenerative diseases (NDDs) [5,20,21,22,23]. Overall, these findings highlight the importance of studying drugs directed to control microglial activation and neuroinflammation in order to assess neuroprotection. 

In addition to increased neuroinflammation, the cholinergic system also exerts decreased functionality during ageing and neurodegeneration. This is of particular interest in NDDs such as AD, where decreased cholinergic functioning has been related to its characteristic cognitive deficits [24,25]. Nicotinic acetylcholine receptors (nAChRs) are widely expressed throughout the peripheral and central nervous system (PNS and CNS); among them, α7 nAChRs are of interest for their role in neuroprotection. In the brain, neuronal and non-neuronal cells express α7 nAChRs. In the former, they are implicated in neurotransmission [26], whereas their role in glial cells has not been fully elucidated. In the PNS, Wang et al. identified that α7 nAChRs in blood monocytes could be controlling inflammation under vagal stimulation and proposed the “cholinergic anti-inflammatory pathway”, which regulates inflammation in the periphery [27]. This proposal has been deeply studied and there is wide consensus on the role of the α7 receptor in controlling inflammation in the periphery [28]. In line with these findings, efforts have been made to elucidate if a similar endogenous pathway is present in the brain. Indeed, microglia express α7 nAChRs [29] and activation of these receptors provides anti-inflammatory properties in cell cultures. Our group has previously described that microglial α7 nAChRs play a key role in providing neuroprotection against brain ischemia [30]. Moreover, evidence of the anti-inflammatory role of α7 nAChR has also been addressed in vivo, were activation of the receptor showed protective effect in a model of chronic stress via inhibition of TLR4 and microglial activity [31]. All together, these findings point at α7 nAChR as an interesting target for neuroprotection, reviewed in [32,33]. Thus, in this study, we aim to evaluate how central α7 nAChR signaling controls the microglial response upon LPS peripheral challenge in adult and aged animals.

## 2. Results

### 2.1. Peripheral Co-Adminsitration of the α7 nAChR Agonist PNU282987 and LPS Prevents Sickness Behavior in Adult Mice 

**Figure 1:**
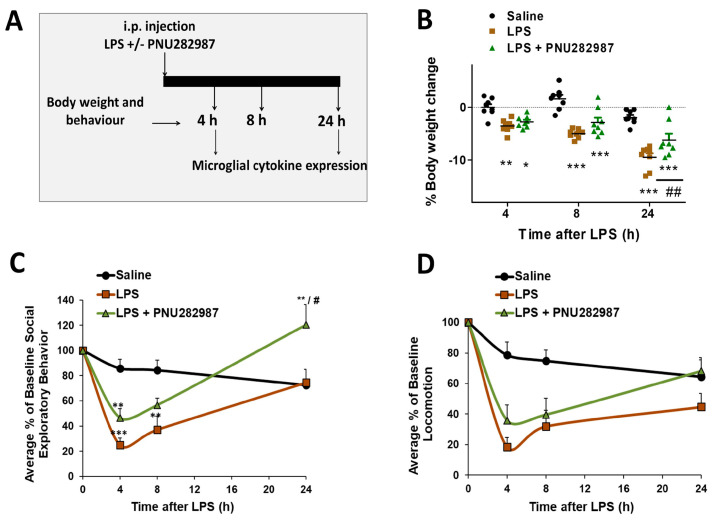
Activation of α7 nAChR by co-administration of PNU282987 and lipopolysaccharide (LPS) via i.p. attenuates behavioral alterations associated with LPS challenge in adult mice. (**A**) The experimental protocol performed in adult Balb/c mice injected i.p. with LPS 0.33 ± 10 mg/kg PNU282987. (**B**) Body weight change, (**C**) locomotor activity and (**D**) social exploratory behavior; measured at 4, 8 and 24 h after injections (Saline, LPS, LPS + PNU282987). Data are represented as the mean and S.E.M (eight animals per group). Comparisons were made using Two-way ANOVA. * *p* < 0.05, ** *p* < 0.01, *** *p* < 0.001 vs. saline; # *p* < 0.05, ## *p* < 0.01 vs. LPS.

To evaluate how microglial α7 nAChR signaling could be controlling neuroinflammation in vivo, we used the LPS-model that drives a transient sickness behavior response characterized by weight loss, decreased social exploratory behavior and loss of locomotor activity. Using this model, we injected the α7 nAChR agonist PNU282987 i.p (10 mg/kg) at the same time as LPS (0.33 mg/kg) and analyzed sickness behaviors at different post-injection time points (0, 4, 8, and 24 h) (Figure 1A). Mice injected with LPS showed progressive body weight loss (4 h: *p* < 0.01; 8 h: *p* < 0.001; 24 h: *p* < 0.001). The body weight loss tended to be attenuated in mice treated with the α7 nAChR agonist at every time-point, although significant differences between LPS and LPS + PNU282987 were only observed 24 h after injection (*p* < 0.01, Figure 1B). Similar results were obtained when sickness behavior was analyzed. LPS injection elicited a marked decrease in locomotor activity (Figure 1C) and social exploratory behavior (Figure 1D), especially 4 and 8 h after injection (Saline vs. LPS *p* < 0.001 and *p* < 0.01, respectively); however, after 24 h, total recovery in social exploratory behavior and a partial recovery in locomotor activity were observed. The decreases in both of these parameters were attenuated when PNU282987 was administered to the mice (Figure 1C,D), although significant differences between LPS and LPS + PNU282987 were only observed in social exploratory behavior 24 h after LPS (LPS vs. LPS + PNU282987 *p* < 0.05)

### 2.2. Activation of α7 nAChRs Prevents Neuroinflammation in Adult Mice Subjected to LPS

**Figure 2:**
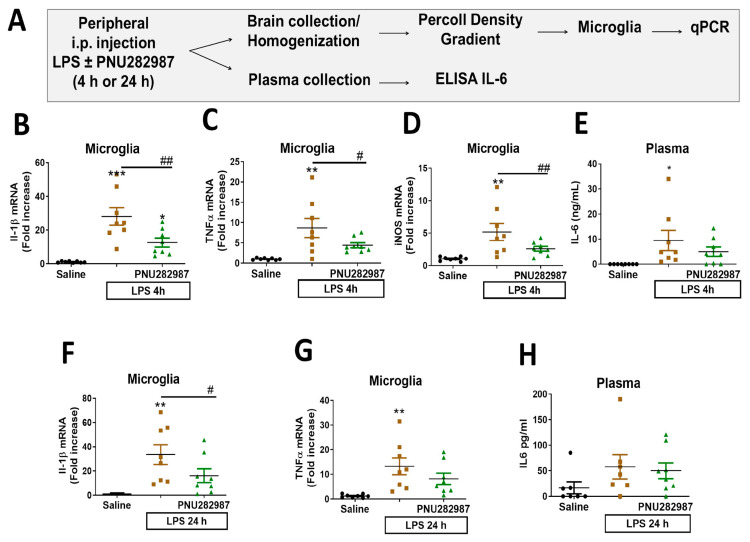
-α7 nAChR activation by co-administration of PNU282987 and LPS via i.p. prevents microglial pro-inflammatory cytokine expression. (**A**) Schematic representation of the experimental procedure used. (**B**) Plasma levels of IL-6 4 h after injections. Microglial mRNA levels of IL-1β (**C**), TNFα (**D**) and iNOS (**E**) 4 h after the different treatments indicated in the X axis. (**F**) Plasma levels of IL-6 24 h after LPS ± PNU282987 injections. Microglial mRNA expression of IL-1β (**G**) and TNFα (**H**) 24 h after injections. Data represent the mean and S.E.M. eight animals per group. Comparisons were made using one-way ANOVA. * *p* < 0.05, ** *p* < 0.01, *** *p* < 0.001 vs. saline; # *p* < 0.05, ## *p* < 0.01 vs. LPS.

This model of transient inflammation is characterized by an acute inflammatory response during the first hours (2 and 4 h post-LPS) where there is a high increase in the expression of pro-inflammatory cytokines. At 24 h post-injection, mice were mostly recovered from sickness behavior, as stated before, although some cytokines remained elevated [34]. Following the experimental protocol described in Figure 1A and Figure 2A, cytokine expression was analyzed at two different time-points: 4 and 24 h post-LPS in plasma and isolated microglia from the brain. 

In isolated microglia, cytokine expression was determined by qPCR (F(3,21) = 22.38). PNU282987 reduced microglial expression of several pro-inflammatory cytokines at the acute phase (4 h post-LPS), including a significant reduction of IL-1β, (*p* < 0.01), TNFα (*p* < 0.05) and iNOS (*p* < 0.01) (Figure 2B–D). Microglial pro-inflammatory cytokine expression was also determined 24 h post-LPS injection (resolution phase) (F(3,21) = 7.867); although iNOS was not increased at this time point (data not shown), PNU282987 reduced the expression of IL-1β (*p* < 0.05) (Figure 2F). Levels of TNFα seemed to be attenuated after treatment with PNU282987, although no statistically significant differences were observed (Figure 2G). Nevertheless, we did observe a significant reduction in TNFα levels after PNU282987 treatment in coronal slices, perhaps due to the lower experimental variability (Figure S1).

Despite the fact that the anti-inflammatory effect of α7 nAChRs in the PNS has been previously reported [35], no statistically significant changes in the expression of plasma IL-6 were observed in mice treated with PNU282987 when compared with LPS-treated mice at any of the time-points studied, 4 and 24 h, (Figure 2E,H), although this result may be related to the high variability in IL-6 plasma levels.

Considering the following factors: (i) the lack of effect on peripheral parameters (plasma IL-6 levels), (ii) the anti-inflammatory effect observed in microglial cytokines and (iii) the permeability of PNU282987 across the blood–brain barrier, the effects elicited by PNU282987 in the present inflammatory model can be attributed to activation of the central α7 nAChR.

### 2.3. Central but Not Peripheral Activation of α7 nAChR Reduces Sickness Behavior and Neuroinflammation Even 2 h after LPS-Challenge in Adult Mice

**Figure 3:**
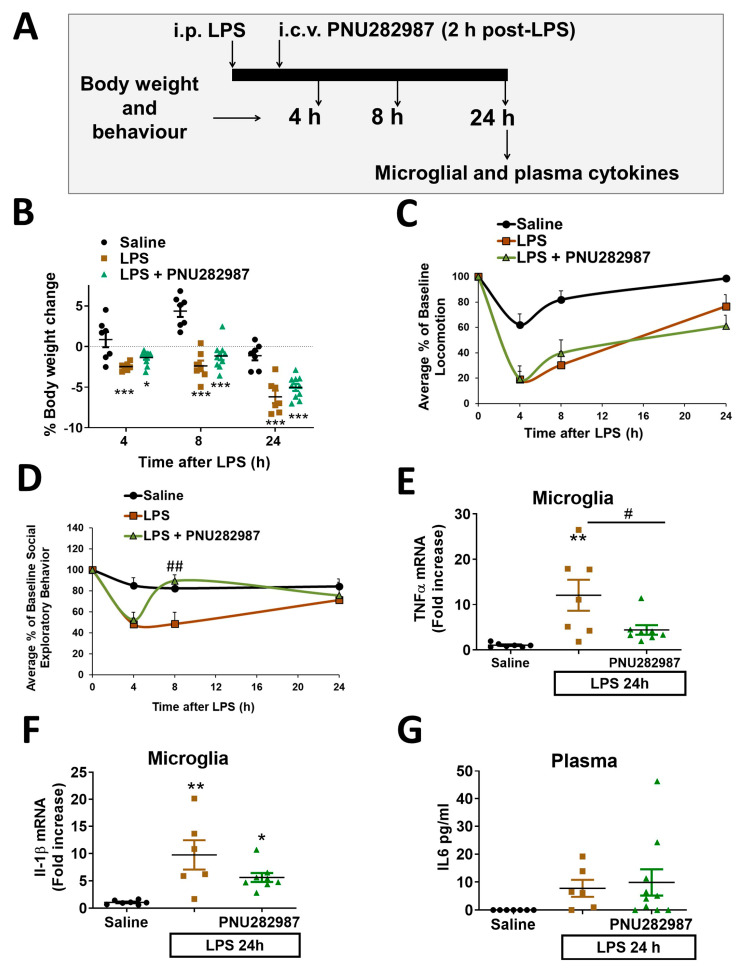
Central activation of α7 nAChRs attenuates sickness behavior and neuroinflammation elicited by LPS challenge in adult mice when administered 2h post-LPS. (**A**) Experimental protocol used in adult Balb/c mice injected i.p. with LPS 0.33 mg/kg ± i.c.v. PNU282987 (10 µg) 2 h post-LPS. (**B**) Body weight change, (**C**) locomotor activity and (**D**) social exploratory behavior; measured 4, 8 and 24 h after saline/LPS injection. (**E**) Plasma IL-6 levels after saline/LPS injections. Microglial mRNA levels of IL-1β (**F**) and TNFα (**G**) 24 h after saline/LPS injections. Data correspond to the mean and S.E.M of 6–8 animals per group. Comparisons were made using one-way ANOVA (**E**–**G**) and two-ways ANOVA (**B**–**D**). * *p* < 0.05, ** *p* < 0.01, *** *p* < 0.001 vs. saline; # *p* < 0.05, ## *p* < 0.01 LPS.

We next sought to determine if the anti-inflammatory effects elicited by PNU282987 administration were due to peripheral or central α7 nAChR activation. For this purpose, we injected the α7 nAChR agonist i.c.v. (intracerebroventricular) to ensure that central receptors were being activated (see experimental protocol represented in Figure 3A). With this protocol, we also determined if PNU282987 could improve recovery once inflammation and sickness behavior were established, as LPS causes acute body weight loss, lethargy and cytokine production after 2 h of its administration [34]. In this case, 10 µg PNU282987 was injected via an i.c.v. cannula 2 h post-LPS. No differences were observed in body weight loss or locomotor activity when comparing LPS and LPS + PNU282987 groups (Figure 3B,C) at any of the times evaluated post-LPS. However, a rapid recovery in social exploratory behavior was observed in mice treated with PNU282987 8 h after-LPS (*p* < 0.01, Figure 3D). 

Mice were sacrificed 24 h post-LPS and pro-inflammatory cytokines in microglia and plasma were determined. PNU282987, centrally administered 2 h post-LPS, reduced the expression of the pro-inflammatory cytokine TNFα (F(3,19) = 7.703, *p* < 0.05) (Figure 3E). IL-1β in microglia showed a tendency to reduction in PNU282987-treated animals, although it did not reach statistical significance (Figure 3F). It should be noted that we did observe a significant reduction in IL-1 β levels after PNU282987 treatment in coronal slices, perhaps due to the lower experimental variability. Furthermore, we corroborated a reduction of TNF-α in coronal slices (Figure S2). As previously observed in the i.p. administration protocol, no changes were observed in plasma levels of IL-6 (Figure 3E)

**Figure 4:**
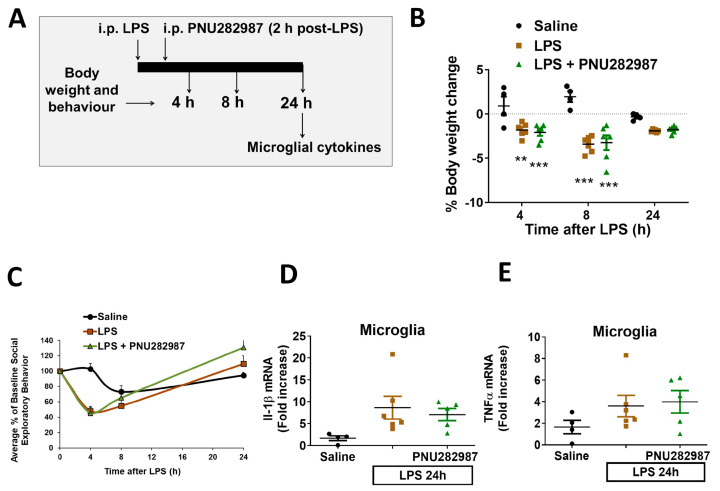
Delayed activation of α7 nAChRs by PNU282987 i.p. administration does not reduce sickness behavior or neuroinflammation induced by LPS. (**A**) Experimental protocol used. Adult (3–4 months old) Balb/c mice were injected i.p. with LPS 0.33 ± 10 mg/kg PNU282987, 2 h post-LPS. (**B**) Body weight change and (**C**) social exploratory behavior measured 4, 8 and 24 h post-treatment (saline, LPS, LPS + PNU282987). Microglial mRNA levels of IL-1β (**D**) and TNFα (**E**) 24 h post-treatments (saline, LPS or LPS + PNU282987). Data are represented as the mean and S.E.M (4–6 animals per group). Comparisons were made using one-way ANOVA (**D**,**E**) and two-ways ANOVA (**B**,**C**). ** *p* < 0.01, *** *p* < 0.001 vs. saline.

This far, α7 nAChR activation prevents certain parameters of sickness behavior and attenuates the neuroinflammatory response when co-administered with LPS peripherally, and recovers those parameters once the inflammation is established when administered centrally. Nevertheless, from a pharmacological point of view, drugs are usually administered peripherally and once the inflammation is established. Thus, we designed a third experimental protocol (Figure 4A) in which PNU282987 was injected i.p. 2 h post-LPS. With this experimental procedure, no improvement, neither in behavior nor in cytokine expression (Figure 4B–D) was observed, indicating that the pharmacological window for the anti-neuroinflammatory effect of α7nAChR activation in this LPS-inflammatory model is shorter than 2 h when the administration is peripheral.

### 2.4. Central α7 nAChR Effects on Sickness Behavior and Neuroinflammation Are Lost in Aged Animals

**Figure 5:**
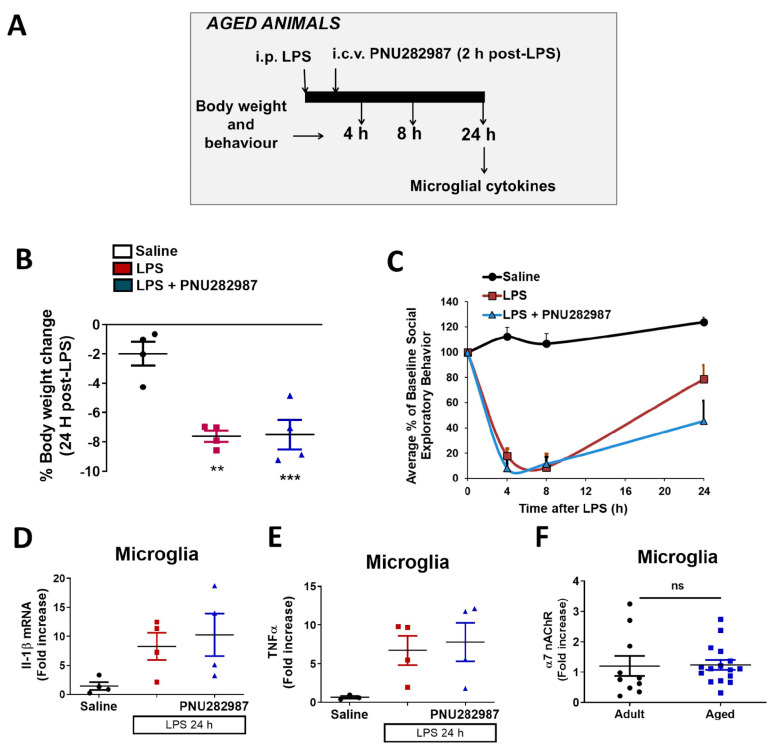
Central activation of α7 nAChRs loses its anti-neuroinflammatory properties in aged mice. (**A**) Experimental protocol used. Aged (18–20 months old) Balb/c mice were injected i.p. with LPS 0.33 mg/kg ± i.c.v. PNU282987 (10 µg) 2 h post-LPS. (**B**) Body weight change, (**C**) social exploratory behavior; measured 4, 8 and 24 h post saline/LPS injection. Microglial mRNA levels of IL-1β (**D**) and TNFα (**E**) 24 h post-treatments (saline, LPS or LPS + PNU282987). (**F**) Levels of α7 nAChR mRNA in adults and aged animals. Data correspond to the mean and S.E.M of 4 animals per group. Comparisons were made using one-way ANOVA (**D**,**E**) and two-ways ANOVA (**B**,**C**). ** *p* < 0.01, *** *p* < 0.001.

Data presented here demonstrate that PNU282987 offers anti-inflammatory effects when administered centrally 2 h post-LPS in adult mice (3–4 months). As microglia become primed with age, we performed the same experimental procedure in aged mice (18–20 months) to determine if PNU282987 conserved its anti-inflammatory effects. As described in Figure 5A, 10 µg PNU282987 were injected using a cannula via i.c.v. 2 h post-LPS and behavior and pro-inflammatory cytokine expression were assessed. Saline or LPS were injected at time 0 and, body weight change and social exploratory behavior were monitored after 4, 8 and 24 h. PNU282987 did not improve neither body weight loss nor the decrease in social exploratory behavior elicited by LPS (Figure 5B,C). As these parameters correlate with the brain inflammatory status, we measured the microglial expression of the pro-inflammatory cytokines IL-1β (Figure 5D) and TNFα (Figure 5E); none of these cytokines were reduced when the mice were treated with PNU282987, indicating that the anti-inflammatory role of α7 nAChR was lost in aged mice. In order to assess if the lack of effect was due to a decrease in the receptor expression during ageing, we measured mRNA α7 nAChR levels, but we did not find any differences when comparing young vs. aged mice (Figure 5F).

## 3. Discussion

The aim of this study was to analyze how microglial α7 nAChR activation controls neuroinflammation caused by LPS in vivo. We have observed that activation of α7 nAChR has a tendency to attenuate sickness behavior and neuroinflammation in adult mice and these anti-neuroinflammatory effects were maintained when administered centrally 2 h after LPS challenge, but not when the agonist was administered peripherally. These results highlight the importance of the route of administration and, also, the therapeutic window to achieve the anti-neuroinflammatory effect. More interesting was the finding that the α7 nAChR anti-inflammatory effect was lost in aged animals and, thus, questions whether the anti-inflammatory effects mediated by α7 nAChRs may be an effective treatment in aged individuals. 

Low-grade chronic inflammatory responses are being recognized in the pathogenesis of most NDDs; therefore, limiting inflammation may be a good strategy to detain disease progression. In this respect, an interesting target to control inflammation is the α7 nAChR that has demonstrated anti-inflammatory and neuroprotective effects in several models of neurodegeneration such as Alzheimer’s [36,37,38] and Parkinson’s disease [39]. Furthermore, in vitro α7 nAChR activation reduced pro-inflammatory cytokine production and had immunomodulatory functions in microglia cultures [29,40,41]. In vivo, nicotine prevents microglial activation or proliferation after LPS injection in substantia nigra [42] and global ischemia in rats [43], and activation of α7 nAChR reduced M1/M2 ratio of macrophages in the peri-infarct of animals subjected to ischemic stroke and bone fracture [44]. These and other studies have provided proof of concept to develop drugs that target this receptor, which have been evaluated in clinical studies for AD [45] and other neurological diseases [46], although with little success.

In spite of all this information, how α7 nAChR signaling controls neuroinflammation has been poorly studied in in vivo inflammatory models. Here, we sought to describe more deeply the specific effect of α7 activation on microglial activation in an in vivo model of inflammation induced by LPS. For that, PNU282987, which selectively activates α7 nAChR [47], was peripherally administered at the same time as LPS and, microglial cytokine expression was evaluated during the acute phase (4 h post-injection) and the resolution phase (24 h post-injection) of inflammation. Overall, we have observed that activation of α7 nAChR has the ability to attenuate most of the pro-inflammatory cytokines in both of the phases evaluated (4 h and 24 h post-LPS), these results correlate with the tendency to improve the behavioral alterations (Figure 1 and Figure 3). These results, together with the fact that PNU282987 crosses the blood–brain barrier [48] raised the question about the implication of central vs. peripheral α7 nAChRs in controlling neuroinflammation. In order to compare the central vs. peripheral effects of α7 nAChR, we injected the agonist either via i.c.v. cannulation or i.p. injection. In both cases, we performed PNU282987 administration 2 h post-LPS, thus we could also assess if α7 nAChR signaling could be a good strategy to control neuroinflammation once the damage is installed. While central administration of PNU282987 2 h post-LPS injection had a positive effect in reducing most of the pro-inflammatory markers (Figure 3), no beneficial effects were observed when PNU282987 was administered peripherally post-LPS challenge (Figure 4). These results point out the importance of microglia vs. macrophages in the anti-neuroinflammatory signaling elicited by α7 nAChR. As stated before, PNU282987 has good brain penetration as presence of the drug in the brain was detected after intravenous injection of the agonist [47]. Nevertheless, we have no data on the active concentration of PNU282987 that reaches the brain after metabolism and/or tissue accumulation and the time-lapse to get to the brain; both issues could explain the lack of effectiveness found when administered i.p, post-LPS injection. In this line, we cannot dimiss that part of the effect could be due to the activation of neuronal α7 nAChRs. Although previous evidence of the group demonstrated that the protection of PNU282987 was lost with the depletion of microglia [30]; however, we cannot exclude that neurons could be, at least in part, modulating microglial response. Moreover, we cannot exclude the effect of PNU282987 on other brain cellular types such as astrocytes, which could also be regulating microglia. In any case, these results highlight the importance of developing drugs that efficiently enter the brain to control neuroinflammation. On the other hand, another interesting observation is the importance of the therapeutic window when targeting the inflammatory response. From our data, it seems that peripheral activation of this cholinergic signaling losses effectiveness once microglia are activated. It must be noted that activation of α7 nAChR 1 h post-ischemia has reported anti-inflammatory effects [30]; however, these results are not comparable to ours because our inflammatory stimulus is much more potent and the agonist was given 2 h (instead of 1 h) post-inflammatory stimuli (stroke vs. LPS). Moreover, the therapeutic window also depends on the agonist and concentration used. For instance, Terrando et al. demonstrated that the α7 nAChR agonist PHA 568487 (0.4 mg/kg i.p.) had anti-neuroinflammatory properties in a model of tibia fracture and endotoxemia when injected 2 h post-insult [48]. Although we do not discard α7 nAChR signaling as a pharmacological target for controlling neuroinflammation, we do highlight the importance of considering the injury model, the therapeutic window and the specific agonist used, as the results may vary.

Finally, there is increasing evidence demonstrating the physiological changes that counteract the ageing process. One of them is the immune system, which is especially altered during ageing. Microglia acquire a so-called “primed phenotype”, characterized by being more pro-inflammatory and resistant to regulation [7,10]. Consequently, during ageing, there is a low grade but constant pro-inflammatory status that is associated with the appearance and progression of neurodegenerative diseases [49,50,51,52]. Moreover, other age-associated changes include adiposity, which may also contribute to the prolonged sickness response after LPS [53]. Thus, it is highly important that pharmacological studies are performed not only in adult but also in aged animals, where the effects can be altered. Here, when we administered the α7 nAChR centrally to aged animals we did not observe any anti-inflammatory effect (Figure 5), contrary to what happened with the adult ones. In light of these results, an open question is whether the loss of α7 immuno-modulation observed in aged animals is due to the primed phenotype described for aged microglia or to a loss in α7 nAChR levels or their function. In this respect, we measured mRNA levels of α7 nAChR but we did not observe differences when we compared adult and aged animals (Figure 5F). These results agree with the general knowledge that cholinergic markers during ageing are mostly unaltered [54]. However, other studies have described a slight decrease in nicotine and α-bungarotoxin (α7 nAChR subunit) binding in humans and rodent brains due to normal ageing [55,56] and, others have reported changes in α7 nAChR mRNA [57]. In any case, what has been nicely described by neurochemical studies is an impairment in cholinergic function following normal ageing [54]. Our results do not show changes in the mRNA expression of α7 nAChR, although we cannot discard that changes could be happening at other levels, such as protein expression, correct sub-cellular localization or function of the receptor. On the other hand, the lack of effectiveness of α7 nAChR stimulation in aged animals could also be due to the primed phenotype that microglia acquire during ageing. Primed microglia have been described to be resistant to regulation by anti-inflammatory signals such as IL-4, IL-10 or TGFβ [7], for instance. During ageing, the endogenous mechanisms controlling neuroinflammation are altered and the protective properties of drugs in adulthood-ageing are not comparable, as we have observed in this study. It should be noted that in this experimental model of LPS challenge, there is robust activation of microglia and although PNU282987 seems to be unable to decrease the primed hyperactive microglia, activation of α7 nAChRs may decrease microglial activation in other ageing disease models with a more modest inflammatory response. In fact, other studies have shown that activation of α7 nAChRs using different agonists could ameliorate cognitive deficits when administered to aged animals [36,58,59]. Nevertheless, it is important to consider that the negative results obtained in aged animals could potentially predict a lack of effectiveness of α7 nAChRs drugs directed to the ageing population, which could explain the negative results obtained to date [46].

## 4. Materials and Methods

### 4.1. Mice

Male adult BALB/c mice (3–4 months old) were obtained from the breeding colony kept in barrier-reared conditions in a specific-pathogen-free facility at the Ohio State University. Aged (18–20 months old) BALB/c mice were purchased from the National Institute of Aging. Mice were housed in polypropylene cages and maintained at 25°, with ad libitum access to water and rodent chow and with 12 h light/12 h dark cycle. All procedures were in accordance with the National Institute of Health Guidelines for the Care and Use of Laboratory Animals and were approved by The Ohio State University Institutional Laboratory Animal Care and Use Committee. All experiments were performed in males to reduce variability. The number of mice depended on the experimental protocol performed: (i) co-administration of PNU282987 and LPS i.p. (8 animals/group), (ii) i.c.v. injection of PNU282987 to adult animals (6–8 animals/group), (iii) 2 h post-LPS administration of PNU282987 i.p. (4–6 animals/group), (iv) i.c.v. injection of PNU282987 to aged animals (4 animals/group).

### 4.2. Animal Body Weight

Animals were weighed before LPS/drug administration (T = 0 h) and at 4, 8 and 24 h post-LPS injection. Results are summarized in Supplementary Table S1.

### 4.3. Intracerebroventricular Cannulation

Intracerebroventricular (i.c.v) cannulation was performed as previously described [60]. Briefly, animals were deeply anesthetized with ketamine (100 mg/kg i.p.) and xylazine (10 mg/kg i.p.), the surgical place was saved and sterilized and the animals were placed in a stereotaxic instrument. An incision of 1.5 cm length was made on the skin and once Bregma was localized, a 26-guage stainless-steel guide cannula was placed in the cerebral ventricle using the following coordinates: Lat 0.5 mm; and A.P 1.2 mm to the Bregma; and Hor-2 mm from the dura mater. Two anchoring cranial screws were inserted adjacent to the cannula and the cannula was secured with cranioplastic cement. A dummy cannula was inserted in the guide cannula to prevent occlusion and infection. For the analgesic, Buprinex was administered (111 μg/kg subcutaneously) following surgery and then again 12 h later. To prevent inclusion or infection, a dummy cannula was inserted in the guide cannula. No procedures were performed before at least 7 days to ensure animals recovery. 

### 4.4. Peripheral and Central Injections

In all experiments, mice were intraperitoneally (i.p.) injected with LPS 0.33 mg/kg (serotype 0127: B8, Sigma, St. Louis, MO, USA) dissolved in sterile PBS. The LPS dose was selected because it drives the transient neuroinflammatory response characterized by pro-inflammatory cytokines production and sickness behavior [8,61]. The saline group was injected with the corresponding amount of PBS. Body weight loss, social exploratory behavior and locomotor activity were assessed at different time-points after LPS injection (0, 4, 8 and 24 h). 

PNU282987 was administered both, i.p and i.c.v. For i.p. injections, PNU282987 was initially dissolved in DMSO (20 mg/mL) and prepared for administration in saline (1 mg/mL); animals were injected with a dose of 10 mg/kg. The dose of PNU282987 was chosen based on previous studies from the group [30] that are in line with the literature [62,63]. For i.c.v administration, 10 µg of PNU282987 was administered in a total volume of 2 µL. As control, the rest of the groups were injected with the vehicle (PBS + the same amount of DMSO used to dissolve PNU282987). Every animal received two injections: Saline or LPS (i.p.) and Vehicle or PNU282987 (i.p. or i.c.v.). 

### 4.5. Social Exploratory Behavior 

All behavior tests were video recorded and manually analyzed afterwards. Social exploratory behavior was determined as a measure of sickness behavior, as described before [8]. For these experiments, a juvenile novel mouse was introduced in the cage during 10 min and behavior was videotaped. The total amount of time that the experimental subject was engaged in social investigation of the juvenile (e.g., anogenital sniffing, trailing) was monitored. The results were normalized with those obtained for the same animal at the time-point 0, which was considered as the 100%.

### 4.6. Locomotor Activity

Animals were maintained in their home cages with a size of 26 × 20 cm and were video-taped for 5 min. For analysis, cages were virtually divided into 8 identical rectangles. The number of crossings was analyzed during the last 3 min. The results were normalized with the number of crossings of the same animal at the time-point 0, which was considered as 100%.

### 4.7. Isolation of Microglia from Mice Brain

Microglia were isolated from whole brain homogenates using Percoll gradient as previously described [15,64]. In brief, brains were homogenized in phosphate buffered saline 1× (PBS, pH 7.4) and disaggregated by passing through a 70 µM cell strainer. Homogenates were centrifuged at 600× *g* for 6 min and cell pellets were re-suspended in isotonic Percoll 70%. Then, a discontinuous Percoll gradient was layered (70%, 50%, 35% and 0%) and the gradient was centrifuged at 2000× *g* for 20 min. Microglia were collected from the interface between 70% and 50% and cells were washed with PBS. Previous characterization demonstrated that approximately 85% of these cells were CD11b + /CD45^low^ [64], and considered as “enriched microglia”.

### 4.8. RNA Isolation and RT-PCR

RNA from enriched microglia was isolated using PrepEase kit (USB, Cleveland, OH, USA). RNA from 1 mm coronal brain slices was isolated using Tri-Reagent protocol (Sigma-Aldrich, St. Louis, MO, USA). The RNA concentration was determined by spectrophotometry (Eppendorf, Enfield, CT, USA) and RNA was reversed transcribed to cDNA. Real time PCR (RT-PCR) performed using the Applied Biosystems Taqman^®^ Gene Expression Assay-on-Demand Gene Expression protocol. Reference cDNA (glyceraldehyde-3-phosphate dehydrogenase (GAPDH)) and target cDNA were amplified simultaneously using an oligonucleotide probe with 5′fluorescent reporter dye (6-FAM). Fluorescence was determined on an ABI PRISM 7300-sequence detection system (Applied Biosystems, Foster City, CA, USA); data were analyzed using the comparative threshold cycle (Ct) method and expressed as fold increase from saline controls.

### 4.9. Quantification of Plasma Levels of IL-6

Levels of IL-6 in plasma were determined using BD OptEIA Mouse IL-6 ELISA according to the manufacturer’s instructions (BD Biosciences, San Jose, CA, USA), as previously described [33]. Briefly, 96-well plates were coated with anti-mouse IL-6 capture antibody at 4 °C overnight. After washing the plate, standards (0–1000 pg/mL) and samples were incubated for 2 h at room temperature (RT). Then, the plate was washed and streptavidin–horseradish peroxidase conjugate was added during 1 h at RT. Thereafter, the plate was washed and incubated with tetramethylbenzidine liquid substrate for 15 min. The reaction was stopped using HCl and absorbance was read at 450 nm using a Synergy HT Plate Reader (Bio-Tek Instruments, Winooski, VT, USA). The assay was sensitive to 10 ng/mL of IL-6. 

### 4.10. Statistical Analysis

GraphPad Software was used for statistical analysis. Data are represented as means ± standard error of the mean (S.E.M.). To determine significant effects, two sets of analyses were performed. First, two-way ANOVA and Bonferroni post hoc test were performed when treatment and time after LPS were both considered as predictor variables. These tests were used when analyzing the effect of the different treatments (Saline, LPS and LPS + PNU282987) on physical variables (body weight loss and sickness behavior) at different time-points (0, 4, 8 and 24 h). Then, one-way ANOVA followed by Newman–keuls post hoc was used when only treatment was used as predictor variable. We selected this analysis when comparing the effect of the different treatments (Saline, LPS and LPS + PNU282987) on cytokine expression. Statistical significance was defined as *p* < 0.05. 

## 5. Conclusions

This study shows that activation of microglial α7 nAChRs mitigates LPS-induced behavioral sickness and inflammatory markers in adult but not in aged animals. However, new experiments need to be developed in order to address whether the loss of the anti-inflammatory effects observed during ageing are due to modifications of α7 nAChR localization/function or to the microglia itself, which should be primed and resistant to regulation. From a pharmacological point of view, the present work highlights the importance of performing more in-depth studies in aged animals in order to determine if α7 nAChR signaling is a valid target to control neuroinflammation in age-related diseases.

## Data Availability

The data presented in this study are available on request from the corresponding author.

## Supplementary Materials

The following are available online, Figure S1: α7 nAChR activation prevents pro-inflammatory cytokine expression in coronal brain slices. Figure S2: Central α7 nAChR activation 2 h post-LPS prevents pro-inflammatory cytokine expression in coronal brain slices. Table S1: Animal body weight.

## Sample Availability

Compounds used in this study are commercially available.
