# Supplementary material for: Central Activation of Alpha7 Nicotinic Signaling Attenuates LPS-Induced Neuroinflammation and Sickness Behavior in Adult but Not in Aged Animals
Source: Molecules. 2021 Apr 7;26(8):2107. doi: 10.3390/molecules26082107 (PMC8067582; doi:10.3390/molecules26082107)
Supplement: Supplementary file 1 [file molecules-26-02107-s001.pdf]

<u>**Supplementrary Material**</u>

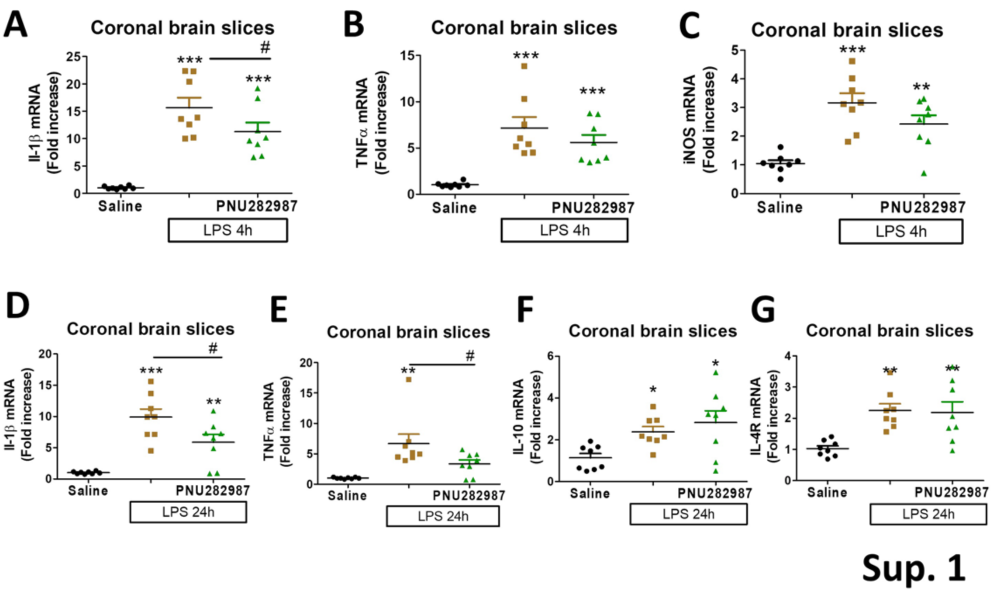

Supplementary Figure S1. α7 nAChR activation prevents pro-inflammatory cytokine expression in coronal brain slices. mRNA levels in coronal brain slices of IL-1β **(A)**, TNFα **(B)** and iNOS **(C)** 4 h after injection of LPS ± PNU282987 and IL-1β **(D)**, TNFα **(E)**, IL-10 **(F),** IL-4R **(G)** 24 h after treatments. Data represent mean ± S.E.M. of 8 animals/group. Comparisons were made using one-way ANOVA. **P*<0.05, ***P*<0.01, ****P*<0.001 *vs* saline; #*P*<0.05 *vs* LPS.

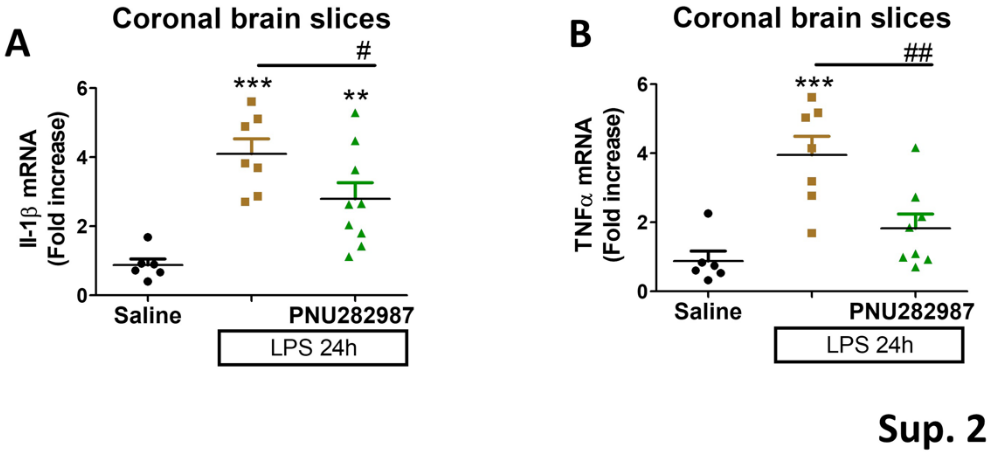

Supplementary Figure S2. Central α7 nAChR activation 2 h post-LPS prevents pro-inflammatory cytokine expression in coronal brain slices. mRNA levels in coronal brain slices of IL-1β **(A)** and TNFα **(B)** were measured 24 h after injection of LPS. Data represent mean ± S.E.M. of 6-8 animals/group. Comparisons were made using one-way ANOVA. ***P*<0.01, ****P*<0.001 *vs* saline; #*P*<0.05, ##*P*<0.01 *vs* LPS.

Supplementary Table S1. Animal body weight. Tables represent mean body weight of animals in the different protocols(A) co-administration of PNU282987-LPS i.p. (8 animals/group), (B) i.c.v. injection of PNU282987 to adult animals (6-8 animals/group), (C) 2 h post-LPS administration of PNU282987 i.p. (4-6 animals/group), (D) i.c.v. injection of PNU282987 to aged animals (4 animals/group). Data correspond to the mean ± S.E.M.

A *Animal body weight: i.p. co-administration of LPS and PNU282987*

	T=0 h	T=4 h	T=8 h	T=24 h
Saline	23.7 ± 0.6	23.7 ± 0.5	24.1 ± 0.5	23.2 ± 0.6
LPS	22.9 ± 0.6	22.1 ± 0.6	21.8 ± 0.6	20.8 ± 0.6
LPS + PNU	24.4 ± 0.5	23.7 ± 0.5	23.7 ±0.6	22.9 ± 0.6

B *Animal body weight: i.c.v. administration of PNU282987 2 h post-LPS (adult animals)*

	T=0 h	T=4 h	T=8 h	T=24 h
Saline	22.7 ± 0.4	22.9 ± 0.4	23.7 ± 0.5	22.4 ± 0.5
LPS	23.1 ± 0.4	22.5 ± 0.4	22.5 ± 0.3	21.6 ± 0.2
LPS + PNU	22.9 ± 0.3	22.5 ± 0.3	22.6 ± 0.3	21.7 ± 0.3

C *Animal body weight: i.p. administration PNU282987 2 h post-LPS*

	T=0 h	T=4 h	T=8 h	T=24 h
Saline	23.6 ± 0.6	23.8 ± 0.5	24.1 ± 0.5	23.3 ± 0.5
LPS	24.0 ± 0.5	23.6 ± 0.6	23.2 ± 0.6	22.1 ± 0.6
LPS + PNU	23.2 ± 0.2	22.8 ± 0.2	22.6 ± 0.4	21.6 ± 0.3

D *Animal body weight: i.c.v. PNU282987 administration 2 h pots-LPS (aged animals)*

	T=0 h	T=4 h	T=8 h	T=24 h
Saline	28.9 ± 1.6	x	x	28.3 ± 1.6
LPS	28.9 ± 1.34	x	x	26.7 ± 0.9
LPS + PNU	29.0 ± 1.2	x	x	26.4 ± 1.1
